# Towards Ecological Sustainability: Assessing Dynamic Total-Factor Ecology Efficiency in Africa

**DOI:** 10.3390/ijerph18179323

**Published:** 2021-09-03

**Authors:** Nelson Amowine, Huaizong Li, Kofi Baah Boamah, Zhixiang Zhou

**Affiliations:** 1 Department of Public Administration and Law, Yibin University, No. 8, St. Luke, Wuliangye, Yibin 644000, China; 2020115001@yibinu.edu.cn; 2 Department of Business and Finance, University of Professional Studies, Accra 23321, Ghana; Kofi.boamah@upsamail.edu.gh; 3 School of Economics, Hefei University of Technology, Hefei 230009, China; zhixiangzhou@hfut.edu.cn

**Keywords:** total-factor ecological efficiency, ecological footprint (EF), human development index (HDI), dynamic meta-frontier SBM, DEA, Africa

## Abstract

Ecological footprint (EF) and human development index (HDI) are two critical indicators for assessing sustainable development worldwide. Past studies in Africa have ignored dynamic sustainable total-factor ecological efficiency (DSTFEE) assessment. This present study proffers a novel dynamic sustainable total-factor ecological efficiency (DSTFEE) that comprehensively assesses the ecological efficiency among 44 sampled African economies from 2010 to 2016. Our study incorporates EF and HDI in the model. Second, the study evaluates regional DSTFEE heterogeneity efficiency as well as the technological gap efficiency in Africa. Further, projection analysis is done to offer a viable solution path to address the inefficient African countries. Third, the study investigates the determinants of ecological efficiency using the bootstrap truncation regression technique. The results from the implemented models are as follows: first, the DSTFEE for the 44 sampled African countries is very low (0.403), indicating enormous potential for improvement. Second, the heterogeneity of DSTFEE across the five Africa regional blocs is evident. The southern bloc had the highest efficiency score, followed by the northern, central, western, and eastern regions. The technology gap ratio also reveals a massive gap among the five Africa regional blocs. Third, the bootstrap truncation regression results established a U-shape nexus between growth and DSTFEE in Africa. REC and trade openness is positively corrected to DSTFEE for African countries. In contrast, financial development, foreign direct investment (FDI), and urbanization impede dynamic ecological efficiency in Africa. The study’s results equip African countries with adequate knowledge of their ecological efficiency situation and provide them a viable path to improve environmental efficiency, thereby boosting their ecological sustainability.

## 1. Introduction

Globally, environmental degradation continues to be a significant challenge that threatens human survival. Environmental degradation is the gradual depletion of the world’s natural resources and has implications for sustainable development [1]. Again, improving the world’s economic output posed a significant threat to environmental quality and contributed enormously to the global climate due to the increasing demand for fossil fuel [2].

The importance of ecological sustainability is well established in the literature. Africa, a renowned continent in the world, is richly endowed with both natural and energy resources. However, the over-exploitation of the continent’s natural resources and other human activities that entail carbon exhaustion has an adverse effect on environmental quality. Hence, the continued increase in African economic output and the growing energy demand ultimately exerts severe ecological challenges. The African population continues to increase, and the energy demand is also increasing [3,4]. This situation has adverse implications for energy use and ecological sustainability. In this context, Alam [5] emphasized that abatement of environmental deterioration is a critical factor in achieving sustainable development.

The sustainable development goal (SDGs) emphasizes the need for countries worldwide to reduce emissions, improve energy use, decarbonize the energy system, and promote the attainment of ecological sustainability [6]. The GDGs advocate for mitigating environmental deterioration, safeguard biodiversity and preserve the entire ecosystem to support inclusive growth. Studies that have investigated the interlinks between the energy-growth-environment for Africa have paid more attention to the impact of growth and energy use on ambient pollutants such as CO_2_ emissions while disregarding sustainable development, which the SDGs have strongly advocated. This study intends to fill these gaps.

This current study aims to investigate the total-factor ecological efficiency in Africa using the novel dynamic sustainable total-factor ecological efficiency (DSTFEE) assessment. This novel model seeks to elucidate comprehensively the sustainable development efficiency across the regions. The study further examines the selected environmental determinants on efficiency for Africa and its regional blocs. The study is concentrated on Africa, as, in the literature, no study has specifically addressed sustainable ecological total-factor efficiency focusing on African countries’ perspectives. Additionally, the study focuses on Africa as a benchmark to adequately offer sufficient contribution to dynamic ecological efficiency from developing African countries.

CO_2_ emission as a measure of environmental degradation has been extensively studied, for instance, among the America [7], European countries [8,9], Asian countries [10,11], as well as recently in Africa countries [12,13,14] at either panel or single country level.

However, using a single pollutant as an environmental degradation index falls short of providing a comprehensive and better picture of the situation since such environmental pollutants only denote a small portion of the environmental degradation [15]. This study adopts the ecological footprint as an index for environmental degradation. This parameter symbolizes the environmental limits and the degree to which humans exceed those limits. As a measure of environmental pollution, ecological footprints give a better indicator as it captures the whole ecosystem that supports humankind’s use and activities. It therefore provides a much more significant picture of environmental deterioration than a single pollutant [16,17].

This study differs from most recent Africa’s efficiency studies [12,13,14]. First, in terms of the input-output selection, this study incorporates both biological resources (ecological footprint (EF)) and the human development index (HDI) factors into the evaluation model [16,17]. Biological resources adequately replaced the energy input factor in the estimated model. The reason for the inclusion of EF is that it gives a better and more profound understanding of environmental degradation than any single pollutant (such as CO_2_ emissions).

Second, according to United Nations Development Programme (UNDP) [18], HDI measures healthy living, the value of human life improvement, and people’s knowledge within a given geographical location. Therefore, HDI is a crucial index that provides an instrumental and objective measure of sustainable development. Unlike prior studies, this study is the first to adopt these two critical, irreplaceable indicators to investigates total-factor ecological efficiency in Africa. The dynamic meta-frontier DEA framework is deployed to measure the STEEE in Africa. Essentially, past efficiency studies in Africa usually neglect the heterogeneity features. Africa has five regional blocs (namely, eastern, western, southern, central, and northern), all of which are different and unique in economic development. Therefore, by integrating Tone and Tsutsui [19]’s and O’Donnell et al. [20]’s meta-frontier, the meta-dynamic SBM model investigates Africa’s sustainable regional ecological efficiency heterogeneity for the first time.

Third, to the best of our knowledge, this study is the first to provide a comprehensive dynamic sustainable total-factor ecological efficiency (DSTFEE) assessment in Africa. The study adopts ecological footprint and HDI indexes as proposed by [16,17] to estimate ecological efficiency in Africa. The computation of the DTFEE index provides a better and more profound understanding of regional environmental efficiency in Africa. This study assesses the sustainable total-factor ecological efficiency of 44 sampled African countries from 2010 to 2016 by incorporating EF and HDI into the estimation model. Furthermore, the heterogeneity of Africa’s sub-regional ecological efficiency is taken into consideration in this study. The estimation of heterogeneity characteristics provides an in-depth understanding of Africa’s different regional blocs’ technological gap.

The remainder of the study is organized as follows. The inclusion of ecological footprint and HDI index into the dynamic meta-frontier DEA model is presented in Section 3. A brief literature review of related studies is discussed in Section 2. The study’s empirical results are shown in Section 4. The conclusions and policy implications of the study are displayed in Section 5.

## 2. Literature Review

Ecological sustainability has become a significant concern for scholars and policymakers mainly due to the emission of greenhouse gases. Environmental efficiency was first proposed by Freeman et al. [21]. Empirically, non-parametric and parametric-based techniques have been employed to measure ecological sustainability efficiency worldwide. Ecological footprint (EF) has been recognized lately as a comprehensive index measuring environmental degradation than any single pollutant.

Currently, ecological sustainability studies focus on these frontiers: total-factor energy efficiency (TFEE), energy efficiency (EE), and total-factor ecological energy efficiency (TFEEE). Energy efficiency depicts the economic benefits (GDP) generated from a per-unit energy consumption [22]. The most commonly used parameter for estimating energy efficiency is per unit economic benefit from energy use, with several practical applications. This kind of single input-single output production frontier was proposed by Patterson [22]. Some scholars argued that since energy is an ecological resource, only energy as an input factor cannot produce economic well-being (GDP). Hu and Wang [23] proposed total-factor energy efficiency (TTEE) to overcome the shortfall. Hu and Wang’s index incorporated labor, capital, and crops cultivated area as inputs factors and employed GDP as the only output factor for estimating the TFEE efficiency of 29 provinces in China from 1995 to 2002. Zhang et al. [24] measured the TFEE of 23 developing economies by including the capital, labor, energy use, inputs, and GDP as the output. Zhao et al. [25] used the Belt and Road Initiative (BRI) economies as objects of the evaluation to study TFEE with the three-stage DEA framework. Hu et al. [26] measured regional TFEE in Taiwan. The above studies typically neglected the incorporation of undesirable output factors. Several scholars incorporated environmental pollutants such as CO_2_ emissions to study TFEE.

Moreover, several scholars believe that only energy input cannot constitute ecological resources. Therefore, the concept of “total-factor ecological efficiency” (TFEEE) was introduced, which considers the ecological footprint index. The inclusion of pollutants as an undesirable output into the framework for evaluating TFEE has been widely studied [27,28,29,30,31]. Yue et al. [32] investigated the TFEEE of G20 economies by utilizing capital, labor, and EF as inputs and economic benefits (GDP) as the outputs factor. From the above, sustainable development efficiency studies demonstrated that estimating ecological efficiency should consist of economic factors and ecological indicators, all within the total-factor production theory framework [31,33].

Furthermore, Lee et al. [34] employed sub-components of energy (coal, oil, and power) and ecological factors to estimate the performance of 27 provinces of China. Cui et al. [35] investigated technical energy efficiency for China’s province based on a three-stage DEA model. In addition, Chaabouni [36] studied energy-saving efficiency in the tourism industry in 31 cities across China, adopting a double bootstrap DEA modeling technique. A study by Shen et al. [16] on regional ecological efficiency is investigated in China using the DEA technique. Shen et al. [17] extended the literature by assessing the ecological development efficiency of the tropics and subtropics in China. For stochastic frontier analysis (SFA), Danish et al. [37] used ecological footprint to study BRICS countries’ economic performance. The impact of renewable and non-renewable energy utilization on the OECD’s ecological footprint was conducted by [38]. The ecological footprint was also included in the studies of [39,40] for MINT and Asian countries, respectively. Nathaniel [41] investigated biocapacity and human capital’s effect on the ecological footprint for G7 economies. Zuo et al. [42] investigated crop production systems in China using the stochastic frontier approach, and their findings provided valuable evidence to support China’s ecosystem improvement. Chen et al. [43] employed the distance function form of the SFA method to assess economic-ecological efficiency in China. They provided paths through which efficiency improvement within the forestry sector in China can be achieved. Gui et al. [44] measured ecological efficiency along China’s Yangtze River economic belt by adopting the SFA framework. In Africa, however, there are several past energy efficiency studies that apply the SFA methodology [45,46,47,48]. Few studies, such as [12,13,14], adopted the DEA framework to assess Africa’s energy efficiency. No study has attempted to measure DTFEEE within the African context and put forward efficiency improvement mechanisms.

Notwithstanding the existing studies on this topic, there are four issues to be addressed. First, in Africa, energy efficiency is measured via fossil energy use, labor, and capital as the input factors, ignoring biological resources or other ecological footprints (grassland, forest land, CO_2_ emissions, and water resources). It is a known fact that sustainable development encompasses: (1) inclusive growth (i.e., resource utilization, society, the economy, and environmental protection), (2) energy efficiency, (3) energy conservation, and (4) energy utility. Related studies typically ignored EF in the African context, and such studies are likely to produce biased results. Second, associated studies in Africa used only GDP as desirable output, ignoring the multidimensionality approach of sustainable development. However, using only the GDP as the desired output indicator has several limitations in comprehensively estimating inclusive growth and the quality of human life within the entire ecosystem [49]. Third, studies investigating ecological efficiency are scant, and the few existing ones are conducted in developed or Asian countries, neglecting the African country’s perspective. Finally, methodologically-wide, the few current ecological efficiency studies adopt the static DEA modeling approach.

To address the first issue, we adopt the EF as an input metric to evaluate Africa’s ecological efficiency. EF is regarded as a comprehensive and collective indicator to reflect environmental degradation than an individual pollutant index [50]. HDI and GDP are utilized as output metrics. At the same time, CO_2_ emission is adopted as a bad output factor to reflect the multiple dimensionality concept of sustainable development to tackle the second issue. The HDI parameter includes a healthy physique, the acquisition of cultural knowledge, and the improvement of the standard of life. Thus, HDI measures human development in a very objective manner [51]. In terms of methodology, this study selected the DEA framework, unlike the SFA, which requires no prior assumption of the production technology. The study utilizes dynamic analysis with fixed assets as a carry-over factor. This helps to understand sustainable ecological efficiency and its comparison among the various Africa regional blocs. Finally, it is abundantly clear that no study has investigated Africa’s ecological efficiency. All the above studies are from developed and Asian economies, where economic development is entirely different and unique.

## 3. Materials and Methods

Integrating Tone and Tsutsui’s [19] dynamic DEA framework and the meta-frontier technique by O’Donnell et al. [20], we built the dynamic DEA model and formulated it as follows.

### 3.1. Dynamic Meta-Frontier DEA Model

African economies are highly heterogeneous, and these differences have been shown in their socio-cultural, economic development, and production structures [36]. The production technologies and management of resources differs across Africa’s regional blocs. African efficiency studies that fail to consider these regional differences are likely to impede policy recommendations. The meta-frontier (MF) DEA concept is adopted in this study to overcomes these shortcomings.

Under different African regional blocks, resources type, and environmental policy, the study assumes that the selected African countries are seen as DMUs $\left(j=1,\ldots ,n\right)$ over T consecutive term $\left(t=1,\ldots T\right)$ in g groups $\left(N=N_{1}+N_{1}\ldots .+N_{G}\right)$. For a year (term), the evaluated African country uses *m* inputs and *s* output factors, respectively. The inputs, desired outputs, and bad output are denoted by $x_{i0t}\left(i=1,2,\ldots m\right)$, $y_{i0t}\left(i=1,2,\ldots s_{1}\right)$ and $y_{iot}^{b}\left(i=1,2,\ldots .s_{2}\right)$, respectively, for each DMU. z is the carry over indicator connecting the periods dynamically.

(1) $$\begin{array}{l} E^{*}=\min\frac{\frac{1}{T}\sum_{t=1}^{T}W^{t}\left[1-\frac{1}{m}\left(\sum_{g=1}^{G}\sum_{i=1}^{m}\frac{w_{i}^{-}s_{it}^{-}}{x_{iot}}\right)\right]}{\frac{1}{T}\sum_{t=1}^{T}W^{t}\left[1+\frac{1}{s_{1}+s_{2}+ngood}\left(\sum_{g=1}^{G}\sum_{i=1}^{s_{1}}\frac{w_{i}^{+}s_{it}^{+}}{y_{iot}}\right)+\sum_{g=1}^{G}\sum_{i=1}^{s_{2}}\frac{w_{i}^{-b}s_{it}^{bad}}{y_{iot}^{bad}}+\sum_{g=1}^{G}\sum_{i=1}^{ngood}\frac{w_{i}^{+}s_{it}^{good}}{z_{iot}^{good}}\right]}\\ s.t.\\ x_{iot}=\sum_{g=1}^{G}\sum_{\partial =1}^{n}x_{ijtg}\lambda_{jg}^{t}+s_{it}\left(i=1,\ldots m,\;t=1,\ldots T\right)\\ y_{iot}=\sum_{g=1}^{G}\sum_{\partial =1}^{n}y_{ijtg}\lambda_{jg}^{t}+s_{it}^{t}\left(i=1,\ldots s_{1},\;t=1,\ldots T\right)\\ y_{iot}^{bad}=\sum_{g=1}^{G}\sum_{\partial =1}^{n}y_{ijtg}^{bad}\lambda_{jg}^{t}-s_{it}^{t}\left(i=1,\ldots s_{2},\;t=1,\ldots T\right)\\ \sum_{g=1}^{G}\sum_{\partial =1}^{n}z_{ijtg}\lambda_{jg}^{t}=\sum_{g=1}^{G}\sum_{\partial =1}^{n}z_{ijtg}\lambda_{jg}^{t+1}\left(vi/t=1,\ldots T-1\right)\\ \sum_{g=1}^{G}\sum_{\partial =1}^{n}\lambda_{jg}^{t}=1\left(t=1,\ldots T\right)\\ \lambda_{jt}\geq 0,\;s_{it}^{-}\geq 0,\;s_{it}^{+}\geq 0,\;s_{it}^{bad}\geq 0 \end{array}$$

### 3.2. The Group-Frontier Model (GFM)

The group efficiency can be accomplished by solving the following model.

(2) $$\begin{array}{l} E_{0}^{*g}=\min\frac{\frac{1}{T}\sum_{t=1}^{T}W^{t}\left[1-\frac{1}{m}\left(\sum_{i=1}^{m}\frac{w_{i}^{-}s_{it}^{-}}{x_{iot}}\right)\right]}{\frac{1}{T}\sum_{t=1}^{T}W^{t}\left[1+\frac{1}{s_{1}+s_{2}+ngood}\left(\sum_{i=1}^{s_{1}}\frac{w_{i}^{+}s_{it}^{+}}{y_{iot}}\right)+\sum_{i=1}^{s_{2}}\frac{w^{-b}s_{it}^{bad}}{y_{iot}^{bad}}+\sum_{i=1}^{ngood}\frac{w^{+}s_{it}^{good}}{z_{iot}^{good}}\right]}\\ s.t.\\ x_{iot}=\sum_{j=1}^{n}x_{ijtg}\lambda_{jt}+s_{it}^{-}\left(i=1,\ldots m,\;t=1,\ldots T\right)\\ y_{iot}=\sum_{j=1}^{n}y_{ijt}\lambda_{jt}^{t}+s_{it}^{+}\left(i=1,\ldots s_{1},\;t=1,\ldots T\right)\\ y_{iot}^{bad}=\sum_{j=1}^{n}y_{ijtg}^{bad}\lambda_{jt}-s_{it}^{bad}\left(i=1,\ldots s_{2}n,\;t=1,\ldots T\right)\\ \sum_{j=1}^{n}z_{ijt}^{\alpha }\lambda_{jt}=\sum_{j=1}^{n}z_{ijt}^{\alpha }\lambda_{j}^{t+1}\left(\forall i;t=1,\ldots ,T-1\right)\\ \sum_{j=1}^{n}\lambda_{jg}^{t}=1\left(t=1,\ldots T\right)\\ \lambda_{jt}\geq 0,\;s_{it}^{-}\geq 0,\;s_{it}^{+}\geq 0,\;s_{it}^{bad}\geq 0 \end{array}$$

### 3.3. Dynamic Technology Gap Ratio Estimation

The computation of the technology gap ratio (TGR) can be accomplished by Equation (3). The TGR is simply the ratio between the meta-frontier relative to the group frontier efficiency. It can be expressed as:

(3) TGR=MFMGFM=E∗EO∗g.

### 3.4. Econometric Model Formulation and Contextual Variables Selection

In this section, the bootstrap truncation regression model is presented. Next, the index selection of the contextual variables and their corresponding hypothesis is introduced.

#### 3.4.1. Regression Technique via Bootstrap Truncation Model

This study analyzes the determinants of economic growth, REC, and some contextual variables on dynamic ecological efficiency in 44 countries in Africa. The study employs the second-stage bootstrap regression by Simar and Wilson [52]. This approach is very robust and can deal with issues of biases and serial correlations [14,53]. The generic form of Simar and Wilson’s regression can be specified as:

(4) $$\phi_{j}=\alpha +\beta Z_{j}+\varepsilon_{j,}\;\;j=1,\ldots ,n,$$

where $\phi_{j}$ denotes the corrected DSTFEE estimates of each understudied country, *j*, obtained from the mathematical model (1). $Z_{j}$ is an index of contextual variables which is anticipated to influence DSTFEE in Africa.

To test the nexus between growth and DSTFEE in Africa, the study presents the following nonlinear model by following prior such [11,54].

(5) $$\begin{array}{l} DTFEE_{it}=\alpha_{i}+\beta_{1}InPGDP_{it}+\beta_{2}{\left(InPGDP_{it}\right)}^{2}+\beta_{3}InREC_{it}+\beta_{4}InUrb_{it}\\ +\beta_{4}InTo_{it}+\beta_{5}InFDI_{it}+\beta_{6}InFDev_{it}+\beta_{7}InIS_{it}+\varepsilon_{it} \end{array}$$

where $DTFEE_{0,t}$ represents Africa’s ecological efficiency estimate obtained from corrected bootstrapped efficiency score. $\alpha_{i}$ and $\varepsilon_{it}$ connotes fixed effect and the error term at time *t*.

#### 3.4.2. Index Selection of Contextual Variables

The nexus between per capita income (PGDP) and ecological efficiency is the Environmental Kuznets Curve. Improvement in income levels at the initial stage of development impedes ecological efficiency. However, after income get to a certain threshold, ecological efficiency improves with further enhancement in income per capita thus, this nexus exhibits a U-shaped curve. Several studies support this hypothesis [11,55]. Moreover, Moutinho et al. [56] investigated the effect of income on efficiency using the super-SBM approach and reported that PGDP has a negative impact on environmental efficiency in Germany. PGDP^2^ is incorporated in the analysis to capture the nonlinear nexus among dynamic ecological efficiency and growth [11]. Therefore, the succeeding hypothesis is valid:

**Hypothesis** **1** **(H1):** *Per capita GDP might have a u-shaped nexus with dynamic ecological efficiency in Africa.*

The rest of the under-investigated variables served as control parameters and are explained as follows:

(1) REC: REC is incorporated in the model, serving as a vital control variable. Clean production technologies such as REC provide the requisite support for CO_2_ emission reduction and conserves energy. Energy is inevitable in African economies, and the utilization of REC innovation will enhance efficiency and lead to CO_2_ emission reduction, which improves the environmental quality [11].

**Hypothesis** **2** **(H2):** *REC might positively affect dynamic ecological efficiency in Africa.*

(2) FDI: FDI offers new and sophisticated technology to the host nation [57] but may result in or transfer polluted industry to the beneficiary nation [58]. Whether FDI significantly aggravates pollution or promotes cleaner technologies needs to be investigated. Therefore, the next hypothesis is valid:

**Hypothesis** **3** **(H3):** *FDI might negatively affect dynamic ecological efficiency in Africa.*

(3) Financial development: can lead to the promotion of ecological degradation in several ways. First, it can help expand business manufacturing processes, which eventually leads to more carbon emissions. Second, it can facilities the attraction of FDI, which may result in more pollution if the host nation has inadequate environmental regulations [59,60]. Therefore, the next hypothesis is valid:

**Hypothesis** **4** **(H4):** *Financial development influences dynamic ecological efficiency in Africa.*

(4) Trade: Farhani et al. [61] and Atta Mills et al. [59] suggested that the over-exploitation of natural resources resulting from trade impedes environmental efficiency. However, Shahbaz and Lean [62] argued that trade offers host countries the advantage to international markets, leading to competition among nations and importing cleaner technologies to reduce CO_2_ emissions. Therefore, the next hypothesis is valid:

**Hypothesis** **5** **(H5):** *Trade openness in African countries significantly impact dynamic ecological efficiency.*

(5) Industrial structure: the development of the industrial structure is a key index to estimate Africa’s economic development. The systemic transformation of this indicator is vital to the development of a green sustainable economy [63]. Therefore, the next hypothesis is valid:

**Hypothesis** **6** **(H6):** *Industrial structure significantly influences dynamic ecological efficiency in Africa.*

(6) Urbanization: In transitional economies like African economies, the labor force, mostly the youth, migrate from the rural to urban cities to seek diverse opportunities. Yasmeen et al. [64] and Kasman and Duman [65] argued that urbanization could lead to environmental degradation. From the above discussion, the succeeding hypothesis hold:

**Hypothesis** **7** **(H7):** *Urbanization might negatively affect dynamic ecological efficiency in Africa.*

### 3.5. Robustness Test via Tobit Regression Model

Borozan [66], Atta Mills et al. [59], and Luo et al. [53] suggested that the Tobit regression should be adopted to perform further robustness tests to confirm the credibility of the initial regression estimates. Therefore, this study employs the Tobit regression technique as a further robustness check tool. The Tobit regression is expressed as:

(6) $$\begin{array}{l} \theta_{o,t}^{*}=\beta_{1}X_{1ot}+\ldots \ldots +\beta_{n}X_{not}+\varepsilon_{ot}\\ \theta_{o,t}=\left\{\begin{array}{l} \theta_{o,t}^{*}\;\mathrm{if}\;0\leq \theta_{o,t}^{*}\leq 1\\ 0\;\;\mathrm{for}\;\mathrm{other}\;\mathrm{values}\;\mathrm{of}\;\theta_{o,t}^{*} \end{array}\right. \end{array}$$

where $\theta_{o,t}$ represents dynamic ecological efficiency of African country o in year t and $\theta_{o,t}^{*}$ is the latent indicator. $X_{ot}\;\mathrm{and}\;\beta$ is the independent variable and regression coefficient, respectively. The error term is denoted by $\varepsilon_{ot}$.

### 3.6. Data and Variables

**Figure 1:**
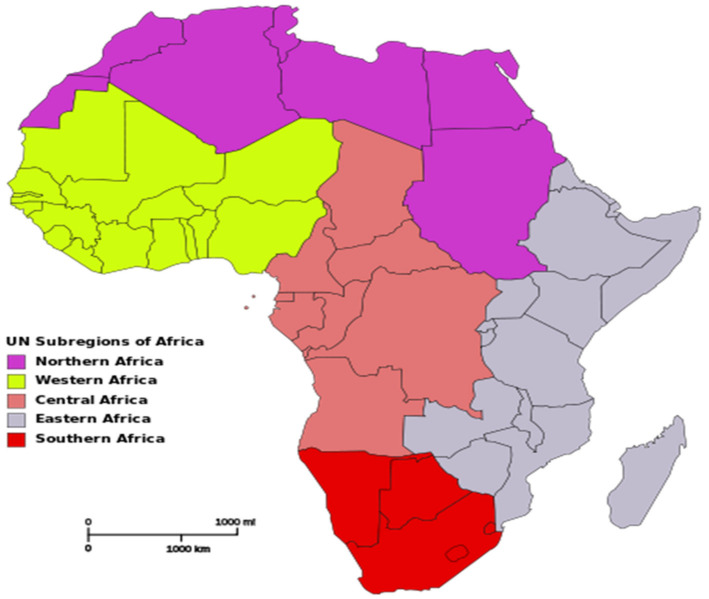
The five sub-regional blocs in Africa.

This study employs panel data from 44 sampled countries in Africa from 2010 to 2016, covering central, northern, western, eastern, and southern African countries. The countries in Africa differ immensely geographically, culturally, socially, and economically. Accordingly, the United Nations division has categorized these countries into five regional blocs, as shown in Figure 1. Because of the non-availability of data, this study selected 4 Southern African countries (namely: Botswana, Lesotho, Namibia, and South Africa); 12 Eastern African countries (Burundi, Comoros, Kenya, Madagascar, Malawi, Mauritius, Mozambique, Rwanda, Tanzania, Uganda, Zambia, Zimbabwe); 8 Central African countries (Angola, Cameroon, Central Africa Republic, Chad, Congo D. R, Congo Rep, Equatorial Guinea, and Gabon); 5 Northern African countries (Algeria, Egypt, Morocco, Sudan, and Tunisia) and 15 west African countries (Benin, Burkina Faso, Cape Verde, Cote d’Ivoire, Gambia, Ghana, Guinea, Guinea-Bissau, Liberia, Mali, Mauritania, Niger, Nigeria, Senegal, and Togo). We were unable to obtain the complete data set for all the 54 African countries. As a result, African countries with missing data in the selected variables were eliminated from the sample. Eventually, the study covers 44 countries in Africa. Data from 2010 to 2016 are sourced from the world development indicators and Global footprint networks. Table 1 shows the definition of the variable used.

As shown in Table 2, the average value of economic growth in the north is the highest of other regional blocs in Africa. This may be due to the fact northern African countries are richly endowed with oil resources. Again, the mean values of labor and EF inputs in the north are far higher than the main panel and the other regional blocs in Africa. Moreover, the consumption of these inputs appeared to have translated into more economic growth for northern African countries. Central and the southern regions had the lowest average labor input consumption in Africa. In terms of fixed assets, the north and south have more fixed assets accumulation relative to the other regional blocs in Africa. From Table 2, the mean values of CO_2_ emissions in the south (3.371) and north (2.116) are the highest, indicating that all African countries must institute CO_2_ emission control policies in their request for inclusive green growth.

Meanwhile, the HDI in the north and south is the highest, while the west recorded the lowest average HDI (0.471). According to the United Nations (UN) definition, an HDI score greater than 0.8 is “very high,” and 0.7 is “high” in human development. Africa with an average HDI of (0.532) falls below the UN’s standards, and there is a need for improvement to achieve sustainable development. Given the increasing populations in Africa, the average EF per person needs to improve significantly to meet international standards. The statistics show that the five regional blocs in Africa are different and highly heterogeneous.

From Table 2, the standard deviations are relatively high relative to the means for inputs, output, and carry-over variables, implying that Africa’s regional bloc varies in size. The high standard deviation values further suggest that a robust check should be conducted on the dataset before application. Data normalization was done on all inputs, outputs, and carry-over indicators, except for HDI and CO_2_ emissions parameters. The second reason that necessitated data normalization is that the HDI index is already a normalized dataset, and modeling such an index with standard data may lead to biased efficiency estimates. More importantly, Ohene-Asare et al. [14] suggested utilizing a VRS model instead of the CRS model in dealing with such a dataset in Africa. To conserve space, the mean normalization index of the study variables is shown in Table 3.

From Table 4, the mean values of per capita GDP (PGDP) appeared to be higher in the southern, central, and northern parts of Africa, while the western part had the lowest average PGDP. REC utilization and development are more evident in the eastern, central, and western Africa countries. Governments in Africa are advised to improve and encourage REC adoption and utilization to promote inclusive growth. The other control variables showed a similar trend across Africa and its regional blocs. The statistics above covered 44 sampled African countries, and the entire 54 African countries’ data may offer a different picture.

## 4. Results

### 4.1. Meta-Frontier Dynamic Sustainable Total-Factor Ecological Efficiency (MDSTFEE) in Africa from 2010 to 2016

**Figure 2:**
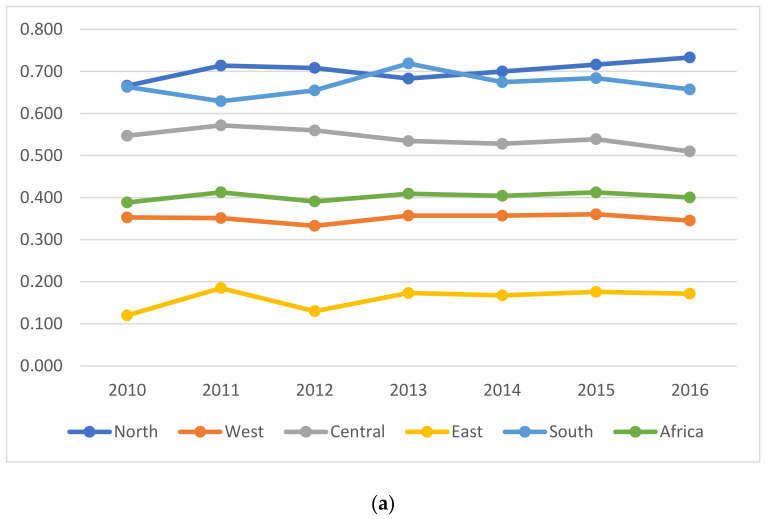

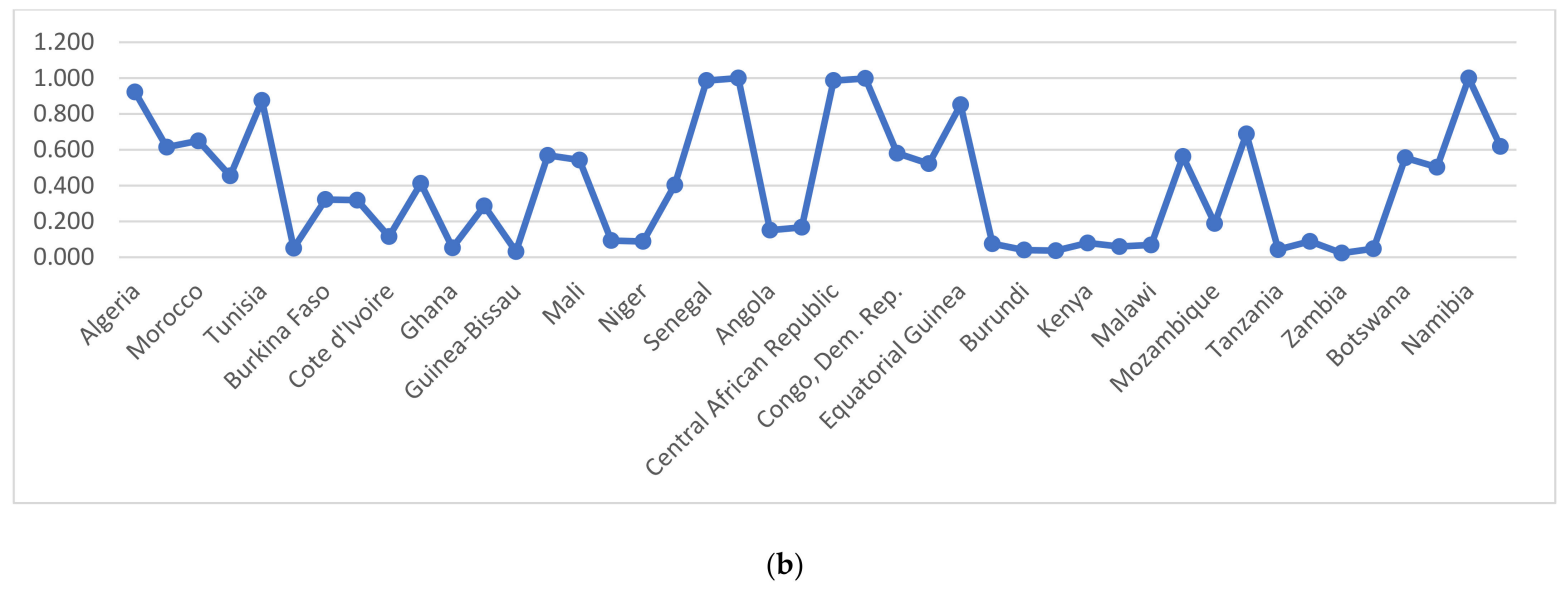
(**a**) Mean total efficiency by Africa blocs. (**b**) Overall mean dynamic ecological efficiency estimates by countries in Africa.

Table 5 shows the overall efficiency from 2010 to 2016. Figure 2a shows entire African mean and annual mean efficiency trends in the five blocs of Africa (i.e., northern, western, central, eastern, and southern Africa regions).

Table 5 and Figure 2b show that Togo and Namibia have overall efficiency estimates of 1 and require no improvement strategies. However, Senegal, Central African Republic, Chad, Algeria, Tunisia, and Equatorial Guinea had slightly encouraging overall efficiency estimates. The rest of the understudied African countries had relatively poor efficiency performances. This implies that anthropogenic activities have a telling effect on efficiency and sustainable development on the African continent.

Figure 2b shows that the overall mean efficiency estimate in the northern region was 0.703, which appeared to be the highest. The southern part followed this with an overall efficiency score of 0.669. The mean efficiency for the central region was 0.541, followed by the western and eastern regions with an overall average efficiency of 0.351 and 0.160, respectively. For the trend analysis, all regions exhibited a downwards trend except for the northern part, which had a downward trend in 2013 but picked up for the rest of the study period.

The mean efficiency for 44 African countries was 0.403 and fluctuated over the period, implying vast room for improvement. From the perspective of sustainable ecological efficiency assessment, this result attests to the conclusion of Shen et al. [16,17] investigating environmental efficiency in China.

The finding shows that carry-over factors, undesirable ecological output (CO_2_ emissions), environmental degradation (EF), and human development index have implications on sustainable ecological efficiency in Africa. Thus, reducing undesirable ecological output, ecological degradation, and the prudent utilization of other input variables will enhance ecological efficiency in Africa.

### 4.2. Group Dynamic Sustainable Total-Factor Ecological Efficiency (GMDSTFEE) in Africa from 2010 to 2016

**Figure 3:**
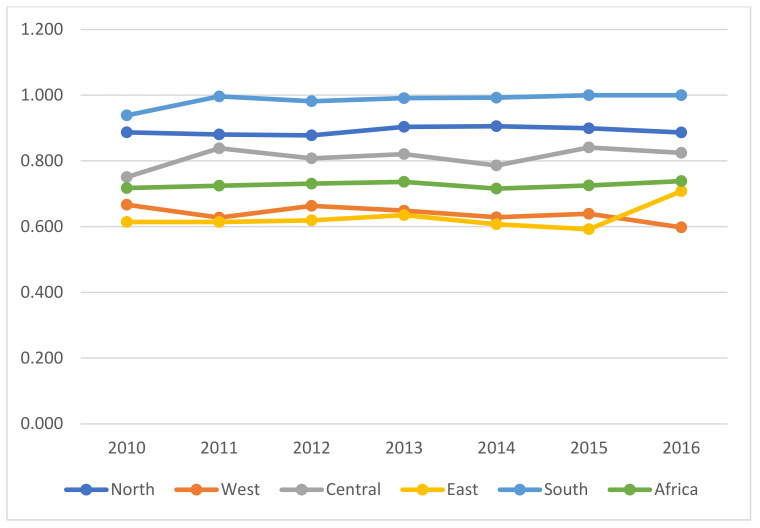
Annual mean trends of GDSTFEE across the five regions in Africa (2010–2016).

Table 6 and Figure 3 show the annual mean regional (group) index efficiencies. The mean trends of GDSTFE index efficiencies in the northern, western, central, eastern, southern, and pool (Africa) from 2010 to 2015. The mean annual efficiencies were efficient in Morocco and Tunisia for the northern. Senegal and Comoros were efficient for the western and central regions, respectively. Furthermore, Algeria (0.99), Egypt (0.927), Benin (0.959), Chad (0.938), and Togo (0.819) remarkedly had the slightly best performances. The rest of the countries had poor ecological efficiency values, showing a need for improvement.

As shown in Figure 3, from 2010 to 2016, the southern region had an overall efficiency of 0.986, indicating that the region was best at this group level. Next, the mean overall efficiency in the northern region was 0.891—the second best. This is closely followed by the central region, with an overall efficiency of 0.810. The western and the eastern regions had 0.639 and 0.627 average overall efficiencies, respectively. From 2010 to 2016, except for the southern region, all the other regions showed a downward growth trajectory.

The mean annual overall efficiency across the five regional blocs in Africa was 0.727, implying the overall pool (Africa) efficiency was best at the group level compared to total efficiency in Table 5. Managerially, the room for improvement at this stage is relatively more minor.

### 4.3. Technology Gap Ratio (TGR) Regarding DSTFEE in Africa from 2010 to 2016

**Figure 4:**
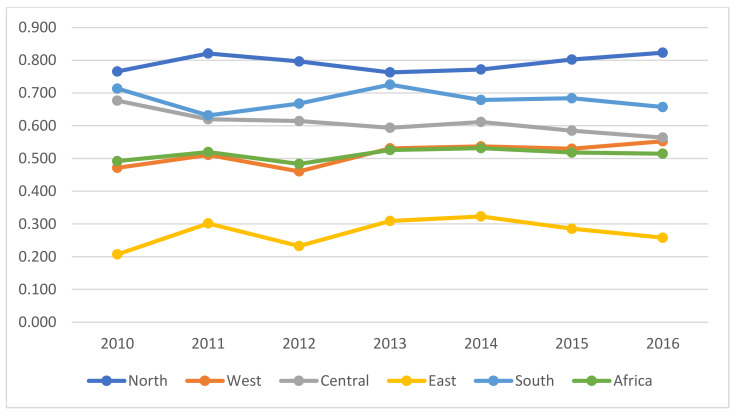
Annual mean trends TGR about DSTFEE in Africa.

The technology gap ratio is essential to the index of the dynamic meta-fronter concept, which is employed to measure the gap of production technology between different regional frontiers. The study adopted Equation (3) to compute the TGR of the five regions in Africa, and the results are shown in Table 7 and Figure 4.

From Table 7, five African countries achieved MTRs equal to 1, 2 in West Africa (Senegal and Togo), 2 in Central Africa (Central African Republic and Chad), and 1 in South Africa (Namibia). However, none of the countries in the northern and eastern parts of Africa showed an MTR estimate of 1. The rest of the African countries investigated were inefficient. According to Figure 4, the mean MTRs of the five regional blocs in Africa are southern, eastern, western, northern, and central. None of these regions have MTRs of 1, indicating a colossal technology gap among these African regional blocs. The northern part of Africa needs to focus more on improving the bloc’s ecological efficiency and tapping the potential for sustainable development. The mean MTR of the north region was (0.792), which implies more room for improvement. The managerial implication here is to narrow the technological gap relative to sustainable development, which is crucial for improving the efficiency of the northern region. The annual mean MTR of the southern, central, western, and eastern Africa was approximately 0.680, 0.609, 0.496, and 0.274, respectively, demonstrating a wide gap among meta- and group frontier in those regions.

### 4.4. Ecological Efficiency Improvements in Africa from 2010 to 2016

Further, the study calculated the projection/adjustment range for selected input and output factors for the inefficient African countries from 2010 to 2016. Such analysis is essential for these countries to achieve total efficiency. The results are shown in Table 8.

From Table 8, the study observes that all African countries’ mean adjustment range estimates present excess of the inputs and shortfall of the output variables. (i) Ecological footprint (a proxy for environmental degradation): From Table 8, EF in African countries required severe reduction by 4.296 on average from 2010 to 2016. Only 2 African countries had efficiency values of 1, implying these countries did not require further adjustment in EF. In addition, Algeria did not require adjustment in EF but was inefficient in other outputs and inputs utilization. African countries can use these two countries as a benchmark to pursue sustainable ecological sustainability—countries such as Benin, Nigeria, Angola, Kenya, Mozambique, Tanzania, and South Africa requires reduction in EF to be efficient. This partly accounted for the lower overall efficiency estimates obtained by these countries in Table 5. (ii) Human development index (HDI): From Table 8, HDI in African countries required an adjustment ratio increased by 0.89 on average from 2010 to 2016. Only 2 African economies (with an efficiency value of 1) did not need adjustment in the HDI indicator. Again, these efficient countries can be utilized as a benchmark for efficiency improvement. Egypt, Tunisia, Benin, Cape Verde, Cote d’Ivoire, Mauritania, Nigeria, Cameroon, Lesotho, and South Africa are among the worst and thus require the most improvement in HDI to be efficient. African countries are advised to take concrete steps towards improving HDI to ensure sustainable development on the continent. (iii) Ecological undesirable output factor (CO_2_ emissions): On average, African countries need to reduce their CO_2_ emissions by 3.39 to improve efficiency. Togo and Namibia required no projection adjustment since their projection values were zero. The rest of the African countries need to cut back on their CO_2_ emissions to be efficient. CO_2_ emission is a serious environmental issue that requires the collaboration of all nations. African countries are advised to take the collective fight against climate change seriously. CO_2_ emission reduction mechanisms must be incorporated into national and regional planning strategies. The consumption of renewable energies will not only enhance ecological efficiency but promote a carbon-free society in Africa. Therefore, these countries are advised to reduce CO_2_ emissions significantly to be efficient next time.

The study also computes the projection difference for fixed assets (carry-over factor) as estimated by the dynamic SBM model. If the projection value is negative, fixed assets (capital) should be reduced to boost efficiency. If the adjustment value is positive, it is indicative that fixed assets should be increased to promote total efficiency. The dynamic DEA model employed in this study aims to measure each African country’s carry-over inefficiency over multiple periods. The incorporation of fixed assets in this study serves as the connecting factor for input and output indicators and reflects the dynamic impact of environmental factors of production. The selection of the fixed asset as a carry-over was based on the orientation or economic basis for African economies to be linked to the next years’ time period, hence, using the fixed asset as a carry-over. Li et al. [69] argued that fixed asset stock is a significant indicator of environmental efficiency and should be adopted as a carry-over indicator to identify the performance gap among decision-making units. Inspired by Li et al. [69,70] and Hsieh et al. [71], the study adopts fixed assets as a carry-over indicator. As shown in Table 8, fixed assets adjustment values are negative for all investigated African countries except for Togo and Namibia’s efficient countries. The average adjustment value of the carry-over inefficiency is (−1.68), implying a reduction of fixed assets in African countries. The worst performers in this category are Egypt, Morocco, Sudan, Tunisia, Mauritania, Cameroon, Chad, Tanzania, Equatorial Guinea, and South Africa, indicating that these countries require huge improvement in fixed assets to be efficient. Overall, African countries could achieve total efficiency by improving environmental degradation, reduce ecological undesirable output factors (CO_2_ emissions), enhancing economic growth, and promoting sustainable development.

### 4.5. Determinants of Ecological Efficiency in Africa

The study attempted to examine the influence of economic growth and other determinants on dynamic ecological efficiency in Africa by applying the bootstrap truncation regression model. The results obtained from the regression analysis are presented in Table 9.

As shown in Table 9, per capita GDP (PGDP) is significantly negative and impedes DSTFEE in Africa. In addition, the quadratic term (PGDP) enhances dynamic ecological efficiency in Africa. This indicates a U-shaped nexus between PGDP and DSTFEE in Africa. This finding is supported by [72]. The effect of growth on ecological efficiency is negative at the initial stages of development to a given point or “critical point” surpassing this mark; its impact improves ecological efficiency. Thus, the relationship between growth and ecological efficiency is dynamic, meaning it can impede or promote based on the level of development. When income levels improve, people are normally conscious about their well-being (health) and demand a clean environment with strict environmental regulations, eventually improving ecological efficiency in some countries in Africa.

REC is positively corrected to dynamic ecological efficiency for Africa. REC utilization is renowned for its significant positive impact on the ecology, simply because of its low carbon nature. As a result, its utilization reduces CO_2_ emissions and enhances ecological efficiency in Africa. These findings support the results of [11] investigating the environmental efficiency and its determinant in Asia-Pacific.

Urbanization is significantly negative and impedes DSTFEE in Africa. These findings suggest that improving urbanization will significantly lead to ecological pollution. The gradual expansion in the urban population, energy demand and transportation aggravate the externality of environmental hazards in urban Africa. The study findings are similar to the results obtained by [64], investigating the nexus between environmental regulation, innovation, and urbanization in China.

The industrial structure positively correlates to dynamic ecological efficiency and insignificantly influences ecological efficiency in African countries. Although industrial structure does not influence dynamic ecological efficiency in Africa in this study, it is important to emphasize that African economies’ pursuit of industrialization must incorporate environmental protectionisms policies to achieve sustainable development. In addition, newly developed industrialized countries are highly dependent on fossil fuel consumption rather than ecological sustainability, which leads to environmental degradation [73].

Trade openness is positively corrected to dynamic ecological efficiency in Africa. This indicates that trade can significantly influence ecological efficiency in Africa. Our findings support [74] results for China.

From Table 9, we observed that financial development is negatively correlated to dynamic ecological efficiency in Africa. Our findings support the results of [75]. These findings suggest that regulators of the financial sector in Africa must develop innovative mechanisms to develop the financial sector in Africa. A sophisticated financial sector can significantly stimulate the channeling of funds towards an ecological-friendly industry by investing in green technologies and environmental projects in African countries.

Foreign direct investment is negatively and significantly related to ecological efficiency in African countries. This implies that FDI deteriorates ecological efficiency since it could provide a pollution intensity industry. These findings are supported by [74] for China. Notwithstanding these findings in Africa, FDI offers the host country the opportunity for advanced technology [57] but may pollute the host nation’s industry [58], as suggested by this study’s findings.

### 4.6. Robustness Check Analysis in Africa

To confirm the validity and credibility of the above findings, we further conduct a robustness check by changing the evaluation method. Atta Mills et al. [59] and Luo et al. [53] provided valuable justification of why the Tobit model’s results should be used as a robustness tool. Inspired by these prior studies, this study also employs the Tobit model to perform further robustness checks on the results in Table 9. We present the results of the Tobit model in Table 9. The signs of the coefficient of key explanatory indicators (*InGDP*, *InREC*) do not change in Table 9, which demonstrates the reliability of the study’s conclusions.

## 5. Conclusions

Environmental degradation has a dire implication on human survival on Earth through worsening biodiversity. In the Anthropocene era, human activities deplete nature and pose threats to survival. One way to ensure ecological sustainability and help African countries towards sustainable development is to boost ecological efficiency assessment. Currently, African countries face severe environmental degradation and little progress toward achieving sustainable development goals. As a way to promote green growth, ecological efficiency integrates economic well-being with environmental protection. It is a viable way to meet the 2030 sustainable development policy drive.

The study substituted EF for the frequently used energy input. HDI, and GDP serves as the desired output factors and CO_2_ emissions is used as undesirable output in the proposed DSTFEE in Africa. To account for the heterogeneity of regional technologies in Africa, the dynamic meta-frontier SBM-DEA model was employed to assess the STFEE of 5 regional blocs in Africa from 2010 to 2016. Most efficiency studies on the continent used a single pollutant for environmental degradation. However, EF gives a comprehensive and bigger picture of environmental degradation than a single pollutant(s). Again, Human Development Index (HDI) was incorporated as a desirable output to give a comprehensive and clear picture of sustainable development in Africa than the only GDP. Further, the study computed the projection analysis to adequately aid the inefficient African countries to improve their relative ecological efficiency. The truncated regression technique discusses the determinants of ecological efficiency.

The study’s main findings are as follows. First, by considering the dynamic meta-frontier technique incorporating ecological undesirable output factor (CO_2_ emissions), the overall mean ecological efficiency of the 44 sampled African countries is low (0.403) from 2010 to 2016. This implies that, on the whole, Africa’s sustainable ecological efficiency was not high during the study period. Regionally, the DSTFEE of the southern bloc was the highest, followed by the northern, central, western, and eastern regions. This indicates the presence of substantial regional gaps in the STFEE of the regional blocs in Africa.

Further, the TGRs result shows a significant gap between the Africa regional blocs. The northern part was the highest efficiency estimates, followed by the southern, central, western, and eastern regions. Third, two African countries (Togo and Namibia) required no projection adjustment in their input/output factors, given that their projection values were zero. These African countries were able to manage the balance between economic well-being and environmental degradation. The rest of the selected African countries need to adjust their inputs/outputs to achieve sustainable development on the continent.

In the second stage, the study examines the determinants of dynamic ecological efficiency adopting bootstrap truncation regression technique, and the main indicates are growth and REC. The findings suggest a U-shaped nexus between growth and DSTFEE in Africa. REC is positively corrected to DSTFEE for African countries. The other control variables exhibited a certain degree of influence on ecological efficiency in Africa.

The study’s results suggest several policy implications that can help governments and policymakers of the sampled African countries curtail the adverse effects of environmental degradation. To improve ecological efficiency, the governments need to direct their efforts towards sustainable development. The use of the only GDP as a measure of economic prosperity must be looked at again. The use of the GDP index to evaluate economic well-being hamper environmental governance and the sustainable development drive of these countries. Therefore, sustainable policies should focus on improving ecological efficiency in Africa. Adopting HDI as an output factor in measuring ecological efficiency in Africa encourages these countries to boost sustainable development on the continent.

Second, the regional difference can be narrow by leveraging economic and technological advantages, set an example for sustainable ecological efficiency improvement in all regional blocs in Africa, reducing CO_2_ emissions, and improve resource management efficiency. Moreover, Africa is mainly a resource endowment continent. The regional economies intensely depend on these resources, and the over-exploitation encourages biocapacity deterioration, deforestation, CO_2_ emissions, and increasing EF. Therefore, one viable way of attaining ecological sustainability and abating EF and CO_2_ emissions is to adopt sustainable practices in the natural resource sector and boost low-polluting energy sources’ utilization.

Policymakers and government authorities can mitigate the adverse impacts of environmental degradation by reducing CO_2_ emissions on the continent and transiting to REC development and utilization. Admittingly, only depending on REC will not be easy because most African countries are low-income nations. However, the shareholders can start by implementing palliative mechanisms such as tax waivers and a tax rebate for the household interested in REC. This will serve as succor and motivate households to boost REC utilization.

The study is relevant. It proffers recommendations for implementing strategies to accomplish the “Paris Agreement” and “Kyoto Protocol”, emphasizing environment quality and creating awareness on climate change and its implications. There are several global perspectives to this research, and it is super essential to development partners and scholars outside of Africa. Countries are anticipated to conform to the “Paris Agreement” stipulations by limiting the utilization of fossil fuels and reducing environmental pollutions.

## Figures and Tables

**Table 1:** Variable definitions.

Variables	Abbreviation	Definition	Source
Labor	Labor	Labor force (millions of workers)	WDI
Ecological Footprint	EE	Measured by the sum total of croplands, forest, fishing, grazing, CO_2_ emissions, and infrastructure footprints	GFN
Fixed assets	FA	Millions of US dollars	WDI
Gross Domestic Product	GDP	Millions of US dollars	WDI
CO_2_ emissions	CO_2_	Metric tons	WDI
Human development index	HDI	-	GFN
Economic growth (per capita GDP)	PGDP	Constant 2010 US$	WDI
Renewable energy consumption	REC	Percentage (%) of total final energy	WDI
Urbanization	Urb	Urban population (% of total population)	WDI
Trade openness	TO	Trade (% of GDP)	WDI
Foreign direct investment	FDI	Foreign direct investment, net inflows (% of GDP)	WDI
Financial development	FD	Domestic credit to the private sector (% of GDP)	WDI
Industrial structure	IS	Industry value added (% of GDP)	WDI

Note: WDI and GFN represent the World Development Indicators [67] and Global Footprint Networks [68], respectively.

**Table 2:** Summary (descriptive) statistics the output, carry-over, and input indicators in Africa.

Africa’s Regional Blocs		Labor	EF	Fixed Assets	GDP	HDI	CO_2_ Emissions
North Africa	Mean	11,000,000	7.81 × 10^7^	3.77 × 10^10^	1.25 × 10^11^	0.654	2.116
	Std. dev.	1.00 × 10^7^	5.35 × 10^7^	2.80 × 10^10^	7.35 × 10^10^	0.095	1.008
	Min	886,436	2.26 × 10^7^	1.07 × 10^10^	4.32 × 10^10^	0.46	0.391
	Max	3.00 × 10^7^	1.80 × 10^8^	1.03 × 10^11^	2.61 × 10^11^	0.75	3.674
West Africa	Mean	7,877,946	2.88 × 10^7^	6.64 × 10^9^	3.84 × 10^10^	0.471	0.399
	Std. dev.	1.29 × 10^7^	4.83 × 10^7^	1.63 × 10^10^	1.03 × 10^11^	0.072	0.250
	Min	195,394	773,229	6.42 × 10^7^	8.50 × 10^8^	0.32	0.082
	Max	5.53 × 10^7^	2.07 × 10^8^	7.42 × 10^10^	4.62 × 10^11^	0.65	1.143
Central Africa	Mean	6,716,081	1.99 × 10^7^	1.52 × 10^10^	2.70 × 10^10^	0.542	1.723
	Std. dev.	7,939,636	2.91 × 10^7^	2.73 × 10^10^	2.79 × 10^10^	0.130	2.683
	Min	358,501	870,103	1.57 × 10^8^	1.49 × 10^9^	0.34	0.026
	Max	2.70 × 10^7^	1.03 × 10^8^	1.03 × 10^11^	1.05 × 10^11^	0.75	10.089
East Africa	Mean	8,948,341	1.82 × 10^7^	4.61 × 10^9^	1.76 × 10^10^	0.521	0.461
	Std. dev.	6,717,202	1.39 × 10^7^	4.32 × 10^9^	1.44 × 10^10^	0.095	0.813
	Min	171,254	699,873.9	1.65 × 10^8^	9.08 × 10^8^	0.4	0.037
	Max	2.47 × 10^7^	5.05 × 10^7^	1.83 × 10^10^	5.54 × 10^10^	0.79	3.198
South Africa	Mean	6,095,439	4.95 × 10^7^	2.32 × 10^10^	1.08 × 10^11^	0.620	3.371
	Std. dev.	8,453,226	7.88 × 10^7^	3.51 × 10^10^	1.73 × 10^11^	0.082	2.794
	Min	762,845	2,750,643	6.45 × 10^8^	2.27 × 10^9^	0.42	1.082
	Max	2.21 × 10^7^	1.92 × 10^8^	8.95 × 10^10^	4.20 × 10^11^	0.71	8.300
Africa (pool)	Mean	8,153,666	3.18 × 10^7^	1.27 × 10^10^	4.69 × 10^10^	0.532	1.122
	Std. dev.	9,943,247	4.70 × 10^7^	2.31 × 10^10^	9.17 × 10^10^	0.112	1.796
	Min	171,254	699,873.9	6.42 × 10^7^	8.50 × 10^8^	0.32	0.026
	Max	5.53 × 10^7^	2.07 × 10^8^	1.03 × 10^11^	4.62 × 10^11^	0.79	10.089

**Table 3:** Mean normalization of the summary statistics across the regions in Africa.

Variables	Africa	North	West	Central	East	South
labor (input)	1.000	1.352	0.967	0.824	1.097	0.747
EF (input)	1.000	2.458	0.906	0.625	0.574	1.560
Fixed assets (carry-over)	0.9996	2.977	0.524	1.192	0.363	1.836
GDP (desired output)	0.973	2.670	0.818	0.575	0.375	2.315
HDI (desired output)	0.532	0.654	0.471	0.542	0.521	0.620
CO_2_ emissions (bad output)	1.122	2.116	0.399	1.723	0.461	3.371

**Table 4:** Summary statistics of the determinants of DSTFEE across the regions in Africa.

Variables	Africa	North	West	Central	East	South
DSTFEE	0.403	0.703	0.351	0.541	0.160	0.669
Economic growth (PGDP)	2493.761	3284.929	1196.570	4403.956	1514.049	5488.011
REC	58.841	18.768	65.539	67.986	70.177	31.511
Urbanization	43.098	54.635	43.497	54.980	27.564	50.022
Trade openness	71.501	61.859	71.027	79.148	61.010	101.504
FDI	5.184	2.029	7.391	4.008	5.242	3.032
Financial development	26.315	44.690	19.652	10.738	24.229	65.745
Industrial structure	26.155	28.889	21.425	41.840	19.396	29.385

**Table 5:** Africa’s MDSTFEE overall efficiencies from 2010 to 2016.

Regions	Countries	2010	2011	2012	2013	2014	2015	2016	Mean
North Africa	Algeria	1	1	1	0.909	0.846	0.839	0.860	0.922
	Egypt, Arab Rep.	0.555	0.630	0.599	0.608	0.623	0.652	0.634	0.615
	Morocco	0.511	0.737	0.712	0.533	0.636	0.646	0.772	0.650
	Sudan	0.508	0.490	0.377	0.462	0.443	0.462	0.436	0.454
	Tunisia	0.756	0.712	0.853	0.903	0.952	0.982	0.964	0.875
West Africa	Benin	0.042	0.067	0.047	0.053	0.048	0.047	0.052	0.051
	Burkina Faso	0.053	0.396	0.319	0.342	0.384	0.410	0.348	0.322
	Cape Verde	0.493	0.498	0.267	0.241	0.241	0.245	0.243	0.319
	Cote d’Ivoire	0.059	0.043	0.136	0.144	0.141	0.144	0.138	0.115
	Gambia, The	0.423	0.411	0.410	0.402	0.401	0.412	0.428	0.412
	Ghana	0.057	0.055	0.052	0.055	0.051	0.049	0.048	0.052
	Guinea	0.049	0.041	0.223	0.398	0.382	0.429	0.482	0.286
	Guinea-Bissau	0.044	0.038	0.027	0.025	0.024	0.025	0.039	0.032
	Liberia	0.886	0.566	0.513	0.544	0.553	0.518	0.402	0.569
	Mali	0.621	0.554	0.435	0.574	0.549	0.547	0.520	0.543
	Mauritania	0.096	0.083	0.090	0.094	0.094	0.097	0.099	0.093
	Niger	0.064	0.098	0.098	0.0875	0.0781	0.095	0.096	0.088
	Nigeria	0.408	0.421	0.413	0.4003	0.410	0.391	0.382	0.404
	Senegal	1	1	1	1	1	1	0.903	0.986
	Togo	1	1	1	1	1	1	1	1
Central Africa	Angola	0.153	0.174	0.198	0.177	0.145	0.104	0.106	0.151
	Cameroon	0.063	0.228	0.184	0.158	0.177	0.185	0.175	0.167
	Central African Republic	1	1	1	1	1	1	0.903	0.986
	Chad	0.986	1	1	1	1	1	1	0.998
	Congo, Dem. Rep.	0.649	0.602	0.613	0.556	0.556	0.560	0.527	0.581
	Congo, Rep.	0.675	0.488	0.443	0.502	0.496	0.547	0.501	0.522
	Equatorial Guinea	0.775	1	1	0.803	0.773	0.825	0.780	0.851
	Gabon	0.074	0.083	0.042	0.079	0.077	0.090	0.084	0.076
East Africa	Burundi	0.055	0.061	0.020	0.042	0.036	0.033	0.034	0.040
	Comoros	0.037	0.043	0.050	0.039	0.040	0.023	0.027	0.037
	Kenya	0.092	0.075	0.080	0.084	0.075	0.078	0.072	0.079
	Madagascar	0.070	0.063	0.051	0.061	0.057	0.060	0.056	0.060
	Malawi	0.032	0.034	0.056	0.089	0.082	0.087	0.099	0.068
	Mauritius	0.035	0.651	0.440	0.724	0.673	0.715	0.702	0.563
	Mozambique	0.037	0.394	0.186	0.160	0.169	0.171	0.198	0.188
	Rwanda	0.875	0.700	0.488	0.683	0.674	0.739	0.658	0.688
	Tanzania	0.042	0.042	0.055	0.035	0.040	0.043	0.042	0.043
	Uganda	0.085	0.082	0.074	0.088	0.098	0.095	0.097	0.089
	Zambia	0.025	0.026	0.020	0.023	0.022	0.021	0.023	0.023
	Zimbabwe	0.056	0.048	0.039	0.049	0.043	0.046	0.052	0.048
South Africa	Botswana	0.385	0.570	0.642	0.713	0.555	0.527	0.492	0.555
	Lesotho	0.621	0.521	0.517	0.448	0.440	0.499	0.473	0.503
	Namibia	1	1	1	1	1	1	1	1
	South Africa	0.647	0.427	0.461	0.715	0.703	0.709	0.663	0.618
	Mean	0.389	0.413	0.391	0.409	0.404	0.412	0.400	0.403

**Table 6:** GMDSTFEE in Africa from 2010 to 2016.

Regions	Countries	2010	2011	2012	2013	2014	2015	2016	Mean
North Africa	Algeria	1	1	1	1	1	0.979	0.946	0.99
	Egypt, Arab Rep.	0.894	0.880	0.877	0.970	0.954	0.982	0.935	0.927
	Morocco	1	1	1	1	1	1	1	1
	Sudan	0.540	0.522	0.513	0.548	0.573	0.536	0.551	0.541
	Tunisia	1	1	1	1	1	1	1	1
West Africa	Benin	0.956	0.890	0.876	0.991	1.000	1.000	1.000	0.959
	Burkina Faso	0.512	0.605	0.582	0.464	0.550	0.564	0.644	0.560
	Cape Verde	0.577	0.515	0.480	0.477	0.482	0.497	0.445	0.496
	Cote d’Ivoire	0.814	0.781	0.730	0.740	0.593	0.516	0.566	0.677
	Gambia, The	0.636	0.659	0.591	0.559	0.518	0.519	0.466	0.564
	Ghana	0.533	0.389	0.388	0.428	0.395	0.411	0.346	0.413
	Guinea	0.112	0.102	0.985	0.937	0.899	0.965	0.826	0.689
	Guinea-Bissau	0.185	0.153	0.120	0.148	0.106	0.106	0.109	0.133
	Liberia	1	1	1	1	1	1	0.955	0.994
	Mali	0.702	0.643	0.650	0.679	0.705	0.669	0.575	0.661
	Mauritania	0.556	0.490	0.415	0.526	0.469	0.506	0.376	0.477
	Niger	0.602	0.614	0.585	0.624	0.556	0.574	0.467	0.575
	Nigeria	0.819	0.570	0.545	0.486	0.487	0.545	0.511	0.566
	Senegal	1	1	1	1	1	1	1	1
	Togo	1	1	1	0.667	0.667	0.712	0.683	0.819
Central Africa	Angola	0.455	0.793	0.726	0.730	0.725	0.840	0.814	0.726
	Cameroon	0.629	0.643	0.625	0.716	0.628	0.681	0.653	0.653
	Central African Republic	0.674	1	0.964	0.980	0.948	1	1	0.938
	Chad	1	1	1	1	1	1	0.955	0.994
	Congo, Dem. Rep.	0.706	0.879	0.837	0.722	0.647	0.875	0.857	0.789
	Congo, Rep.	1	1	1	1	1	1	1	1
	Equatorial Guinea	1	1	1	1	1	1	1	1
	Gabon	0.543	0.392	0.310	0.417	0.342	0.331	0.318	0.379
East Africa	Burundi	0.309	0.289	0.107	0.109	0.106	0.134	0.121	0.168
	Comoros	1	1	1	1	1	1	1	1
	Kenya	0.930	0.910	0.837	0.988	0.957	0.513	0.699	0.834
	Madagascar	0.645	0.598	0.604	0.614	0.613	0.127	0.127	0.476
	Malawi	0.801	1	1	1	1	1	1	0.972
	Mauritius	1	1	1	1	1	1	0.931	0.9516
	Mozambique	0.497	0.785	0.705	0.581	0.530	0.662	0.500	0.609
	Rwanda	0.994	0.930	0.963	0.866	0.785	0.891	0.958	0.912
	Tanzania	0.320	0.111	0.109	0.117	0.111	0.121	0.897	0.255
	Uganda	0.123	0.186	0.155	0.110	0.107	0.565	0.576	0.260
	Zambia	0.365	0.371	0.477	0.673	0.496	0.474	0.954	0.544
	Zimbabwe	0.385	0.459	0.473	0.562	0.582	0.617	0.732	0.544
South Africa	Botswana	1	0.985	0.926	0.963	0.970	1	1	0.978
	Lesotho	0.755	1	1	1	1	1	1	0.965
	Namibia	1	1	1	1	1	1	1	1
	South Africa	1	1	1	1	1	1	1	1
	Mean	0.717	0.724	0.731	0.736	0.716	0.725	0.738	0.727

**Table 7:** Technology gap ratio (TGR) regarding DSTFEE in Africa.

Regions	Countries	2010	2011	2012	2013	2014	2015	2016	Mean
North Africa	Algeria	1	1	1	0.909	0.846	0.858	0.909	0.932
	Egypt, Arab Rep.	0.620	0.716	0.683	0.627	0.653	0.665	0.678	0.663
	Morocco	0.511	0.737	0.712	0.533	0.636	0.646	0.772	0.650
	Sudan	0.941	0.939	0.735	0.843	0.772	0.861	0.792	0.840
	Tunisia	0.756	0.712	0.853	0.903	0.952	0.982	0.964	0.875
West Africa	Benin	0.044	0.075	0.053	0.053	0.048	0.047	0.052	0.053
	Burkina Faso	0.103	0.655	0.548	0.738	0.698	0.727	0.541	0.573
	Cape Verde	0.855	0.967	0.557	0.505	0.500	0.493	0.547	0.632
	Cote d’Ivoire	0.072	0.055	0.186	0.195	0.238	0.279	0.244	0.181
	Gambia, The	0.664	0.624	0.693	0.719	0.775	0.793	0.919	0.741
	Ghana	0.108	0.141	0.134	0.129	0.128	0.118	0.140	0.128
	Guinea	0.442	0.401	0.227	0.424	0.425	0.444	0.584	0.421
	Guinea-Bissau	0.236	0.251	0.221	0.169	0.228	0.232	0.357	0.242
	Liberia	0.886	0.566	0.513	0.544	0.553	0.518	0.421	0.571
	Mali	0.884	0.862	0.668	0.844	0.779	0.817	0.904	0.823
	Mauritania	0.172	0.169	0.216	0.179	0.201	0.191	0.265	0.199
	Niger	0.106	0.160	0.167	0.140	0.141	0.165	0.205	0.155
	Nigeria	0.498	0.739	0.758	0.823	0.842	0.718	0.748	0.732
	Senegal	1	1	1	1	1	1	1	1
	Togo	1	1	1	1	1	1	1	1
Central Africa	Angola	0.336	0.219	0.272	0.243	0.200	0.123	0.130	0.218
	Cameroon	0.101	0.355	0.294	0.220	0.281	0.272	0.269	0.256
	Central African Republic	1	1	1	1	1	1	1	1
	Chad	1	1	1	1	1	1	1	1
	Congo, Dem. Rep.	0.920	0.685	0.732	0.770	0.861	0.640	0.616	0.746
	Congo, Rep.	0.675	0.488	0.443	0.502	0.496	0.547	0.501	0.522
	Equatorial Guinea	0.775	1.000	1.000	0.803	0.773	0.825	0.780	0.851
	Gabon	0.137	0.212	0.137	0.190	0.227	0.272	0.265	0.206
East Africa	Burundi	0.178	0.210	0.188	0.380	0.343	0.249	0.282	0.262
	Comoros	0.037	0.043	0.050	0.039	0.040	0.023	0.027	0.037
	Kenya	0.099	0.082	0.096	0.085	0.079	0.151	0.103	0.099
	Madagascar	0.108	0.105	0.085	0.100	0.093	0.470	0.437	0.200
	Malawi	0.040	0.034	0.056	0.089	0.082	0.087	0.099	0.070
	Mauritius	0.035	0.892	0.440	0.724	0.673	0.715	0.753	0.604
	Mozambique	0.075	0.502	0.264	0.276	0.320	0.258	0.396	0.299
	Rwanda	0.881	0.753	0.506	0.790	0.859	0.829	0.686	0.758
	Tanzania	0.130	0.379	0.503	0.298	0.364	0.355	0.047	0.297
	Uganda	0.688	0.442	0.479	0.806	0.909	0.169	0.169	0.523
	Zambia	0.068	0.070	0.043	0.035	0.044	0.045	0.024	0.047
	Zimbabwe	0.146	0.105	0.083	0.087	0.074	0.074	0.070	0.091
South Africa	Botswana	0.385	0.578	0.693	0.740	0.572	0.527	0.492	0.570
	Lesotho	0.822	0.521	0.517	0.448	0.440	0.499	0.473	0.531
	Namibia	1	1	1	1	1	1	1	1
	South Africa	0.647	0.427	0.461	0.715	0.703	0.709	0.663	0.618
	Mean	0.492	0.520	0.483	0.526	0.532	0.518	0.515	0.512

**Table 8:** Mean adjustment range for selected indicators in Africa.

Regions	Countries	EF	HDI	Fixed Assets	CO_2_ Emissions
North Africa	Algeria	0	0.38	−0.61	−1.01
	Egypt, Arab Rep.	−1.80	1.52	−5.71	−10.61
	Morocco	−3.05	1.12	−4.13	−3.45
	Sudan	−9.98	1.10	−4.57	−1.94
	Tunisia	−9.45	1.75	−4.47	−15.73
West Africa	Benin	−10.03	1.14	−2.31	−2.17
	Burkina Faso	−3.19	0.97	−1.18	−0.85
	Cape Verde	−0.21	1.27	−0.29	−3.11
	Cote d’Ivoire	−3.57	1.37	−2.10	−4.00
	Gambia, The	−0.01	0.88	−0.01	−0.40
	Ghana	−4.98	0.68	−1.30	−0.87
	Guinea	−0.15	0.13	−0.03	−0.09
	Guinea-Bissau	−1.43	0.96	−0.08	−0.56
	Liberia	−0.10	0.57	−0.02	−0.21
	Mali	−1.80	0.63	−0.46	−0.56
	Mauritania	−1.71	1.73	−6.41	−9.57
	Niger	−1.47	1.00	−1.24	−1.34
	Nigeria	−40.10	1.69	−2.35	−16.06
	Senegal	−2.82	0.16	−0.07	−0.68
	Togo	0	0	0	0
Central Africa	Angola	−22.15	1.09	−1.76	−0.41
	Cameroon	−4.11	1.80	−5.38	−8.87
	Central African Republic	−0.03	0.56	−0.05	−0.04
	Chad	−2.81	0.26	−10.39	−0.32
	Congo, Dem. Rep.	−0.25	1.05	−1.06	−0.13
	Congo, Rep.	−0.09	0.90	−1.86	−8.08
	Equatorial Guinea	−0.18	0.91	−2.21	−7.15
	Gabon	−1.39	0.89	−1.35	−1.16
East Africa	Burundi	−0.85	0.69	−0.12	−0.28
	Comoros	−0.09	0.88	−0.12	−3.19
	Kenya	−3.39	0.93	−0.91	−2.12
	Madagascar	−1.29	1.01	−0.44	−0.96
	Malawi	−0.93	0.89	−0.15	−0.27
	Mauritius	−0.14	0.97	−0.25	−6.96
	Mozambique	−7.76	0.57	−0.95	−0.18
	Rwanda	−1.67	0.51	−0.32	−0.03
	Tanzania	−21.52	0.86	−4.06	−0.30
	Uganda	−8.96	0.80	−1.80	−0.08
	Zambia	−0.08	0.74	−0.06	−0.04
	Zimbabwe	−0.72	0.13	−0.13	−0.34
South Africa	Botswana	−0.98	0.85	−1.41	−4.96
	Lesotho	−0.43	1.24	−0.37	−6.82
	Namibia	0	0	0	0
	South Africa	−13.34	1.47	−1.39	−22.82
	Mean	−4.30	0.89	−1.68	−3.38

**Table 9:** Results of determinants of dynamic ecological efficiency in Africa.

a: Truncated Bootstrapped Regression Result		b: Robustness Check Result via Tobit Regression	
DSTFEE	Coefficient	DSTFEE	Coefficient
InPGDP	−0.0459 *** (0.00498)	InPGDP	−0.1019 *** (0.0063)
InPGDP2	0.01112 *** (0.000458)	InPGDP2	0.01459 *** (0.00047)
InREC	0.00658 *** (0.00109)	InREC	0.01162 *** (0.00161)
InFDI	−0.00298 ** (0.00112)	InFDI	−0.00195 (0.00136)
InFDEV	−0.00413 * (0.00227)	InFDEV	−0.00986 *** (0.00291)
InTRADE	0.01445 *** (0.00439)	InTRADE	0.01914 *** (0.0055)
InIS	0.00096 (0.00313)	InIS	−0.00276 (0.00408)
InURB	−0.00169 ** (0.00387)	InURB	−0.0089 * (0.00469)
Constant	0.5589 *** (0.03108)	Constant	0.89837 *** (0.04004)

Note: In a, the coefficients have been bootstrapped. Standard errors in parentheses. * *p* < 0.10, ** *p* < 0.05, *** *p* < 0.01.

## Data Availability

All data sources are fully disclosed in the body of the manuscript.
